# Women and Children First: The Impact of Sexually Transmitted Infections on Maternal and Child Health

**DOI:** 10.1155/2014/671085

**Published:** 2014-02-18

**Authors:** Consuelo Beck Sagué, Carolyn Black, Stephen A. Morse, George Schmid

**Affiliations:** ^1^Department of Health Promotion and Disease Prevention, Robert Stempel College of Public Health and Social Work, Florida International University, 11200 SW 8 Street HLS (AHC II) 571, Miami, FL 33199, USA; ^2^Centers for Disease Control and Prevention (CDC), Atlanta, GA 30333, USA; ^3^Centers for Disease Control and Prevention (CDC), Astana 010000, Kazakhstan

In 2000, all 189 Member States of the United Nations (UN) adopted the UN Millennium Declaration, committing them to pursue eight measurable targets, the Millennium Development Goals (MDGs) [1, 2] to be achieved by 2015. The MDGs were intended to: (1) eradicate severe poverty and hunger; (2) achieve universal primary education; (3) promote gender equality and empowerment of women; (4) reduce child mortality; (5) improve maternal health; (6) combat HIV/AIDS, malaria, and other infectious diseases; (7) ensure environmental sustainability; and (8) develop a global partnership for development. The unprecedented effort required to achieve the MDGs addresses issues and conditions especially relevant to the health and survival of women—particularly mothers—and infants, children, and youth. Despite calls for universal access to reproductive and sexual health, no MDG addressed these issues, which are critical to maternal and child health and remain neglected [3].

The MDGs proposed dramatic reductions in, or elimination of, scourges that have plagued humankind since its beginnings including severe poverty, famine, and pregnancy-related mortality as well as one emerging infectious disease, human immunodeficiency virus (HIV) infection, only recognized in the last decades of the 20th century, which was singled out for particular attention [1, 2]. Arguably, never before has elimination of the inequalities at the root of these scourges been articulated even as a possibility, let alone as goals to be urgently pursued. But without an MDG focused on reproductive health, progress on this issue relies on its being buoyed by efforts towards addressing the eight MDGs. Progress towards achievement of MDG indicators has been impressive; some (such as the halving of the number of people living in extreme poverty) were achieved before 2015 [4]. Others, including reduction of HIV mother-to-child transmission to less than 2% [5], are either on schedule or achievable by 2016-2017. Much, however, remains to be done.

It is in that context that the disproportionate and largely preventable toll that sexually transmitted infections (STIs) exact on women, including mothers, and infants, children, and youth, is reexamined. Women, particularly adolescent women, are especially vulnerable to STIs due to, among other factors, a larger exposed mucosal surface area, hormonal effects, changes in the protective female genital tract microflora, and the intermittent presence of ectopy, especially in adolescence [6–8]. These groups are also at increased risk due to sexual partnerships with older men, little power over when, where, and how sex occurs, and other social and cultural factors. However, poverty, neglect, and inequality drive much of the increased risk of women and children. The impact of STIs on maternal and child populations is greatest in low- and middle-income countries, where over 75% of STIs reportedly occur [9, 10]. Within these countries and in underserved populations in industrialized countries STIs continue to disproportionately impact the most disadvantaged women and children [11, 12].

This issue of the Journal of Sexually Transmitted Diseases offers heartening news about emerging tools for elimination of the impact of these illnesses and reminders that we struggle against formidable forces. Control of the worst outcomes of STIs in low- and middle-income countries is achievable [13]. Moreover, the elimination of mother-to-child transmission of syphilis and HIV infection is clearly attainable; the global commitment to elimination is based on compelling evidence that their elimination is not only possible, but also cost effective and essential to the health of their mothers [14–16]. But unlike the case with smallpox eradication, as long as HIV and *Treponema pallidum* infections exist in the human population—and they will—the threat of mother-to-child transmission remains a possibility [17]. Similarly, as long as orphaning, poverty, neglect, and abuse drive children and youth to homelessness and life in “the streets” in urban settings worldwide, their vulnerability to coerced and unprotected consensual sex, as well as resultant STIs and their sequelae, will continue to be considerable [18, 19].

The papers in this special issue document the tragic circumstances endured by street children in Ethiopia, the elevated risk of herpes simplex virus type 2 infection among monogamous women in India associated with their husbands' work-related travel, and innovations and challenges in the progress towards congenital syphilis elimination in Haiti and Kenya, and HIV mother-to-child transmission in India. The report from the United Kingdom describes the efforts to reach out to the population of children born to HIV-infected parents who even in high-resource environments, while at extraordinarily high risk of perinatally acquired HIV infection and orphaning, often remain invisible, untested, and underserved.

At first glance, these reports appear to be a potpourri of glimpses of the impact of STIs on the health of mothers, infants, and youth. But, in fact, they illustrate both the complex forces that sustain the persistent problem of STIs in these populations and innovative, multilevel approaches that have already resulted in progress towards elimination. The recommendations set forth in the mixed methods study to support street children in Ethiopia may seem painfully obvious but, worldwide, street children are often viewed as a public nuisance or a law enforcement issue; the tragic stories told in the focus groups illustrate how utterly at the mercy of predatory forces these youth really are [18]. Similarly, creating work opportunities for men in rural communities that do not result in long separations of stable couples may reduce the risk of STIs not only in India, where this risk is particularly well documented, but also worldwide as a critically important aspect of global development efforts [20].

The development and implementation of simple point-of-care testing for syphilis have contributed to the identification of infected women in some of the most challenging environments on earth [21–23]. Similarly, innovative processes for dramatically expanding access to prenatal HIV testing and timely initiation and continuation of combination prenatal antiretroviral therapy are being successfully implemented in the most impacted populations in the world [24, 25]. Nevertheless, it is clear that in efforts to eliminate congenital syphilis there are no “magic bullets.” The cascade from antenatal care availability and use, point-of-care testing, and treatment with benzathine penicillin for the pregnant woman and, ideally, her partner is very effective but often fragile; “systems improvements” that consistently guarantee and monitor response to treatment of 100% of infected women can be elusive. The use of cash incentives to microcredit women's groups based on villagers' antenatal care attendance and provision of mobile health care were associated with a dramatic increase in antenatal care enrollment and, as a result, prenatal HIV testing. In nations with large HIV epidemics, conditional cash transfers [26, 27] may contribute to elimination of mother-to-child transmission. The success of case finding for children of HIV-infected parents in the United Kingdom is encouraging to all who understand that, worldwide, HIV-infected parents are often underserved and face daunting problems, and their children are an often hidden and neglected population. Identifying and providing quality services to HIV-affected families remain challenges that are increasingly recognized and addressed [28–30].

These reports highlight challenges, possible solutions, and inspiring successes that worldwide promise the possibility of eliminating the most devastating consequences of STIs for mothers, children, and youth. The slogan “women and children first” inspired an impressive array of reports describing approaches that may help achieve the MDG vision of a fairer, safer world for mothers, children, and youth.

*Consuelo Beck Sagué* *Consuelo Beck Sagué* *Carolyn Black* *Carolyn Black* *Stephen A. Morse* *Stephen A. Morse* *George Schmid* *George Schmid*
